# Extracellular ATP and P_2_X_7_ receptor exert context-specific immunogenic effects after immunogenic cancer cell death

**DOI:** 10.1038/cddis.2015.411

**Published:** 2016-02-18

**Authors:** A D Garg, D V Krysko, P Vandenabeele, P Agostinis

**Affiliations:** 1 Cell Death Research & Therapy (CDRT) Unit, Department of Cellular and Molecular Medicine, Faculty of Medicine, KU Leuven University of Leuven, Leuven, Belgium; 2 Molecular Signaling and Cell Death Unit, Inflammation Research Center, VIB, Ghent, Belgium; 3 Department of Biomedical Molecular Biology, Ghent University, Ghent, Belgium

*Dear Editor*,

Immunogenic cell death (ICD) facilitates danger signalling-driven trafficking of damage-associated molecular patterns (DAMPs) like extracellular ATP (eATP).^1, 2^ The binding of eATP to P_2_X_7_ receptor triggers immunogenic signalling,^3^ which (along with other factors) converts the dying cancer cells into an effective anticancer vaccine.^3^

Endoplasmic reticulum (ER) stress is central to ICD,^1^ on the basis of which ICD inducers are subdivided into two types,^1^ that is, Type I (e.g., some chemotherapies), which elicit danger signalling through 'collateral' non-lethal ER stress,^1^ and Type II (e.g., hypericin-photodynamic therapy (Hyp-PDT)), which elicit danger signalling via 'focused' lethal ER stress.^1, 4^ Type II and Type I ICD inducers differ on several levels, for example, plasticity of danger signalling and the trafficking mechanisms of DAMPs.^4^ In fact, eATP was found to be absent during Newcastle disease virus (NDV)-induced Type II ICD despite the induction of macroautophagy (a Type I ICD-associated, eATP-trafficking mechanism).^2, 5^ Moreover, we have established that Hyp-PDT-induced eATP is PERK and secretory pathway-dependent,^6^ while being independent of macroautophagy^7^ or chaperone-mediated autophagy.^8^ This raised an important question – like in the case of NDV-induced ICD, could eATP be dispensable or a partial immunogenic signal for Hyp-PDT-induced ICD?

To this end, we decided to gain further insights into the eATP-trafficking mechanism and its immunogenic potential following Hyp-PDT. To address the contribution of the pannexin/connexin-caspase axes^2^ that elicits eATP secretion (in response to Type I ICD inducers but remains enigmatic in the Type II settings), we utilized the pan-pannexin/connexin inhibitor, carbenoxolone (CBX). In CT26 cells treated with Hyp-PDT, CBX pretreatment failed to reduce eATP (Figure 1a), thereby suggesting the dispensability of pannexins/connexins. Next, we addressed the role of caspase activity using the pan-inhibitor, zVAD-fmk. Interestingly, zVAD-fmk significantly reduced Hyp-PDT-induced eATP (Figure 1a). Considering the previously demonstrated role of casp-8 in ICD^1, 6^ we wondered whether this caspase was mediating eATP secretion. Interestingly, CT26 cells expressing caspase-8 shRNA (casp-8 shRNA) also exhibited significantly reduced eATP following Hyp-PDT (Figure 1a).

The regulation of eATP secretion by casp-8 was unexpected, as our previous study found casp-8 to be dispensable for Hyp-PDT-induced ICD, *in vivo*.^6^ This suggested that eATP secretion may not be crucial for Hyp-PDT-induced ICD, *in vivo*. To resolve this, we utilized the CT26-BALB/c mice prophylactic vaccination model. Immunogenic effects of eATP were blocked using either Apyrase or Apy (an ATP-degrading enzyme, Figure 1b) or a 2,3-dialdehyde derivative of ATP, that is, oxidized-ATP (Oxi-ATP, a P_2_X_7_ receptor antagonist) or a combination of both (i.e., Apy+Oxi-ATP).^3^ Approximately 70% of the mice immunized with Hyp-PDT-based vaccine efficiently rejected the formation of CT26 tumours at the challenge site (Figure 1c). Interestingly, eATP degradation or blockade of P_2_X_7_ receptor, alone, failed to strongly reduce the tumour-rejecting immunity (Figure 1c). On the other hand, only the combination of Apy+Oxi-ATP significantly reduced the vaccine's tumour-rejecting capacity (Figure 1c). Thus, eATP, despite being ubiquitously secreted after Hyp-PDT,^6, 7, 8^ only acts as a partial immunogenic signal, and thus singular blockade of either eATP or its P_2_X_7_ receptor is unable to reduce the immunogenic potential of the vaccine.

These results are unprecedented because eATP and P_2_X_7_ receptor had been shown to act in a synergistic manner.^1, 2, 3^ Here, we rather observed a potentiating effect, that is, blockade of either eATP or P_2_X_7_ receptor did not, but combined blockade significantly reduced ICD's immunogenic potential. Thus, our results suggest that the mere presence of eATP does not ensure the presence of corresponding immunogenic activity in all contexts. Moreover, a certain degree of redundancy exists on the level of purinergic receptor agonists, and thus these results may also point to the release of such (as-yet-uncharacterized) agonists from dying cells. Lastly, these observations are based on the heterotopic (subcutaneous) tumour model; it would be crucial to reanalyze the role of eATP in an orthotopic tumour model to overcome immunological variations stemming from incompatibility between the transplanted cancer type and the surrounding tissue.

## Figures and Tables

**Figure 1:**
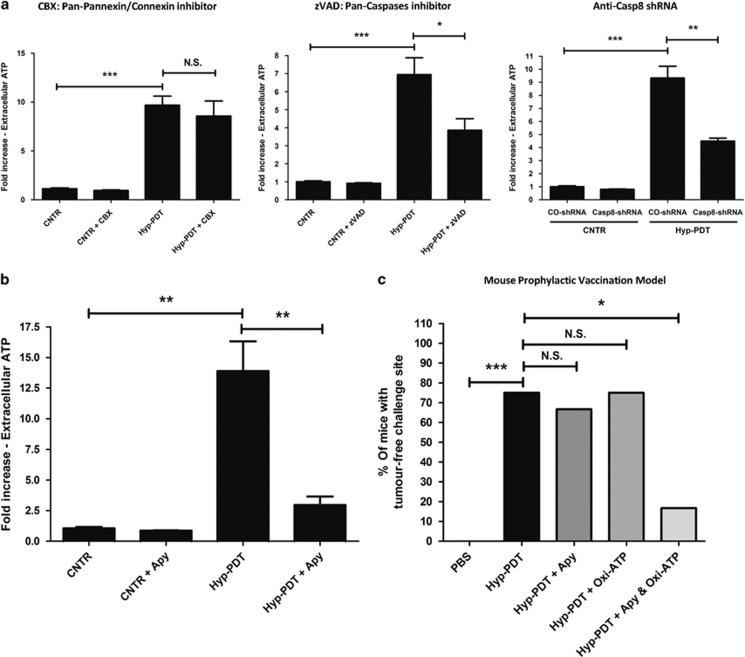
Extracellular ATP and P_2_X_7_ receptor together potentiate ICD in cancer. (**a**) CT26 cells were treated with Hyp-PDT (dosage: 150  nM Hypericin preincubation for 16  h followed by light irradiation with a total fluence of 2.70  J/cm^2^) as described previously^6^ and recovered for eATP analysis 1  h post treatment. Depending on the settings (as indicated in the legends above the graphs), the cells were preincubated with CBX (100  *μ*M for 1 h) or zVAD-fmk (25 *μ*M for 30  min). Alternatively, CT26 cells expressing control shRNA (CO-shRNA) or casp-8 shRNA were utilized as described previously.^6^ Extracellular ATP was detected using the standard luciferin-luciferase bioluminescence assay.^7^ Here, *n*=3–4, mean±S.E.M., Student's *t*-test, **P<0.01 and ***P<0.001, NS, non-significant; CNTR, untreated controls. (**b**) In another case, CT26 cells were treated with Hyp-PDT as described above and incubated for 15  min post recovery with Apyrase (Apy; 10  U/ml); eATP was then analyzed as described above. (**c**) For testing of immunogenicity, the CT26-BALB/c mice model was utilized.^6^ Here, the CT26 cells were treated with Hyp-PDT followed by 'vaccine' preparation as described previously.^6^ In certain cases, the vaccines were mixed/co-injected with either Apy (10  U/ml for 15  min) or Oxi-ATP (4  mg/kg per mouse) or both (Apy+Oxi-ATP). These respective vaccines were given twice with an interval of 7–8 days between vaccinations in one of the flanks of the syngenic BALB/c mice. About 8–10 days following the vaccination regimen, the vaccinated mice were challenged on the contra-lateral flank with live CT26 cells. Thereafter, the mice were monitored for the occurrence of CT26 tumours at the challenge site. Here, *n*=10 for PBS, *n*=12 for Hyp-PDT, *n*=12 for Hyp-PDT+Apy, *n*=12 for Hyp-PDT+Oxi-ATP and *n*=6 for Hyp-PDT+Apy+Oxi-ATP, Fisher's exact test; *P<0.05, **P<0.01 and ***P<0.001; NS, non-significant

## References

[bib1] Garg AD et al. Front Immunol 2015; 6: 588. 26635802 10.3389/fimmu.2015.00588 PMC4653610

[bib2] Martins I et al. Cell Death Differ 2014; 21: 79–91. 23852373 10.1038/cdd.2013.75 PMC3857631

[bib3] Ghiringhelli F et al. Nat Med 2009; 15: 1170–1178. 19767732 10.1038/nm.2028

[bib4] Garg AD et al. Cell Death Differ 2014; 21: 26–38. 23686135 10.1038/cdd.2013.48 PMC3858605

[bib5] Koks CA et al. Int J Cancer 2015; 136: E313–E325. 25208916 10.1002/ijc.29202

[bib6] Garg AD et al. EMBO J 2012; 31: 1062–1079. 22252128 10.1038/emboj.2011.497 PMC3298003

[bib7] Garg AD et al. Autophagy 2013; 9: 1292–1307. 23800749 10.4161/auto.25399

[bib8] Garg AD, Dudek AM, Agostinis P. Cell Death Dis 2013; 4: e826. 24091669 10.1038/cddis.2013.372 PMC3824681

